# Doctor-patient communication skills: a survey on knowledge and practice of Iranian family physicians

**DOI:** 10.1186/s12875-021-01491-z

**Published:** 2021-06-24

**Authors:** Ramin Shiraly, Hamideh Mahdaviazad, Ali Pakdin

**Affiliations:** 1 Department of Community Medicine, School of Medicine, Shiraz University of Medical Sciences, Shiraz, Iran; 2 Health Behavior Science Research Center, Shiraz University of Medical Sciences, Shiraz, Iran; 3 Department of Family Medicine, School of Medicine, Shiraz University of Medical Sciences, P.O. Box: 7193634154, Shiraz, Iran; 4 Student Research Committee, Shiraz University of Medical Sciences, Shiraz, Iran

**Keywords:** Doctor- patient relation, Family physician, Knowledge, Practice

## Abstract

**Background:**

Communication skills are fundamental to successful medical practice and can greatly impact patient satisfaction, compliance and outcomes. This study evaluated knowledge and practice of doctor- patient communication among the urban family physicians based on main items of Calgary Cambridge Observation Guides.

**Method:**

This cross-sectional study was conducted from July to September, 2019, in a 400 randomly selected sample of family physicians of Shiraz, Fars province. The data collection tool was a self-administered, second-part questionnaire developed by the researchers. Participants were asked about their age, gender, practice setting, and years of work experience and if they received any formal training in doctor- patient communication. Data were analyzed using SPSS (Version 16, SPSS Inc., Chicago, IL, USA). A p-value of less than 0.05 was considered statistically significant.

**Results:**

The study participants included 204 male and 196 female family physicians with a mean age of 46.7 ± 7.7 years. The mean communication skills knowledge score was 41.5 (SD: ± 2.8) indicating a high level of knowledge. The mean score for practices was 38.7 (SD: ± 3.4), implying a moderate level of practice. Based on Bloom’s scale, nearly 80% of family physicians had good knowledge about doctor-patient communication skills, however, 55% of participants reported moderate to poor level of practice in this regard. Results of multivariate regression analysis suggest that higher levels of related knowledge, having higher age or longer work experience, and working in the public sector can predict better practice scores (*P *< 0.005).

**Conclusion:**

There is a potential gap between knowledge and self-reported practices toward communication skills among a sample of Iranian family physicians. They have fundamental weakness in the most important evidence-based items of doctor- patient communication. Considering significant role of family physicians in prevention and control of non-communicable diseases (NCDs) as an emerging challenge of our country, the topic of communication skills should be inserted as a top educational priority of family physicians.

## Background

Communication skills are fundamental to successful medical practice and can greatly impact patient satisfaction, compliance and outcomes. Although these skills are dependent on various personal factors, it has been shown that communicative abilities could be enhanced by training and experience [1–3]. Lack of comprehensive training curricula of communication skills for medical professionals and differences in educational styles have led to the development of guidelines such as Calgary-Cambridge guide to the medical interview to provide physicians with evidence-based recommendations for improving their ability to communicate effectively [1, 4–7].

The concept of doctor-patient relationship has evolved over time from a paternalistic model to a patient-centered one which honors patients’ needs, autonomy and preferences. Nowadays, patient-centered approach is becoming more popular in clinical practice. This approach furnish a medium that encourages physicians to get into the patient’s world and see the illness from patient’s perspective” [8–11].

Like many other countries around the world, Iran is facing an increasing burden of NCDs. Family doctors as primary care physicians are the front line of contact for different population groups including patients with common chronic diseases such as diabetes and hypertension and can play an essential role in prevention and control of NCDs through patient education and behavioral change strategies [12, 13]. These aims could be achieved by creating good doctor-patient relationships. Poor quality doctor- patient communication has been found to be associated with patient dissatisfaction, reduced treatment adherence and poor health outcomes [14–19].

Several studies have investigated barriers and promoters for a good doctor-patient relationship [7, 14, 17, 20]. Reports show that most of physicians have little formal training in communication skills and this accountability has not been integrated formally into the curriculum or continuing educations of many medical schools [7, 21].

At the best of our knowledge, there are no studies to evaluate urban family physicians’ awareness or practice regarding doctor- patient communication in Iran. Current study conducted to evaluate knowledge and practice of doctor- patient communication among the family physicians of Shiraz University of Medical Sciences based on main items of Calgary Cambridge Observation Guides.

## Methods

### Study design and participants

This was a cross-sectional study conducted from July to September, 2019, in a randomly selected sample of family physicians of Shiraz, Fars province. All 1120 physicians from the Family Physician Department membership list who practice either in government/public clinics or private offices in Shiraz were targeted as the study population. The required sample size was computed by an online statistical calculator [22] with assuming 50% anticipated prevalence of good knowledge and practice, 95% confidence interval and 5% margin of error. Considering a non-response rate of 10%, the final sample size composed of 427 physicians who were selected by simple random sampling method.

### Ethical considerations

The protocol was approved by the Shiraz University’s Ethics Committee (IR.sums.med.rec.1397.582). Verbal informed consent was obtained from all family physicians, after they had been informed of the study’s goals. Complete anonymity and data confidentiality was guaranteed. The research conducted in this study was performed in accordance with the Declaration of Helsinki.

### Data collection

Data gathering began in consultation with Vice Chancellor’s office; it involved coordination with the family physicians’ workplaces, and obtaining the necessary official permissions. In the next step, supervisors were trained in the distribution and completion of the questionnaires. Participants who did not give consent to participate, or did not attend offices after three visits were excluded from the study.

The data collection tool was a self-administered, second-part questionnaire developed by the researchers. The first part focused on self-reported demographic and professional data; participants were asked about their age, gender (men/ women), practice setting (government/public and private), and years of work experience and if they received any formal training in doctor- patient communication (yes/ no). The second part consisted of 20 knowledge and practice questions, it was based on a review of the relevant literature focusing on Calgary–Cambridge framework on doctor–patient communication [23–25]. The questionnaire was reviewed by an expert panel composed of three community medicine specialists, and one epidemiologist who checked and improved its content validity. Reliability was established in a pretest with 30 participants. Cronbach α coefficient was in acceptable range for knowledge and practice questions, and it took, on average, 10–15 min to complete.

The knowledge assessment section consisted of 10 items, each rated on a 5-point Likert scale (‘strongly agree’, ‘agree’, ‘Neutral’, ‘disagree’ and ‘strongly disagree’) and scores were recorded from 1 to 5. Therefore, total score ranged between10 to 50. Similarly, the practice assessment section consisted of 10 items, each rated on a 5-point Likert scale (‘never’, ‘seldom’, ‘occasionally’, ‘often’ and ‘always’) and scores were recorded from 1 to 5 and total score ranged between10 to 50. Knowledge scores were classified into three levels using Bloom’s Theory [26], which categorizes by percentage based on summed scores: ≤ 60% represented poor knowledge, > 60–80% moderate knowledge, and > 80% a good level of knowledge. Likewise, practice scores were classified into poor (≤ 60%), moderate (> 60–80%) and good level of practices (> 80%).

### Statistical analysis

All data were entered, verified and analyzed using SPSS (Version 16, SPSS Inc., Chicago, IL, USA). Descriptive statistics, including number (%) and means ± standard deviation (SD) were calculated for responses to demographic and professional experience data. Correlation between knowledge and practice scores and numerical data (age and work experience) were assessed using the Pearson rank correlation coefficient. Student t-test was used to measure the associations between knowledge and practice scores and categorical variables (gender and practice setting). We used univariate and multivariate linear regression analysis to identify independent factors associated with family physicians, self-reported practice of communication skills. A p-value of less than 0.05 was considered statistically significant.

## Results

Between July and September 2019, a total of 400 family physicians of Shiraz completed the questionnaire. Nineteen physicians were not available during the data collection period and 8 physicians refused to cooperate (response rate: 93.7%). The demographic and professional data of participants are shown in Table 1. The mean (± SD) age of the respondents was 46.7 ± 7.7, and the sample included 204 male and 196 female doctors. More than two thirds of family physicians were practicing in government clinics (79.5%), and the mean (± SD) work experience was 16.8 ± 7.5 years.

**Table 1:** Demographic and professional characteristics of the participants

Variables	N (%) or Mean ± SD
**Age (years)**	46.69 ± 7.68
**Gender**	
Men	204 (51.0)
Women	196 (49.0)
**Practice setting**	
Governmental /public	318 (79.5)
Private	82 (20.5)
**Work experience** (years)	16.82 ± 7.51
**Total**	400 (100)

The overall mean (± SD) score of knowledge and practice for all participants was 41.5 ± 2.8 and 38.7 ± 3.4, respectively (out of maximum possible score of 50). Based on Bloom’s scale, nearly 80% of family physicians had good knowledge about doctor-patient communication skills, however, most of the participants had moderate scores on practice (55%).

Table 2 summarize the frequency of participants’ responses to knowledge and practice questions. On the knowledge and practice scales, more than 65% of the family physicians were strongly confident about skills such as “greeting to the patient” or “introducing himself/herself to the patient” or “encouraging patient to tell the story of the problem”, while, most of participants were reluctant to choose extreme positive responses in Likert scale on skills such as “dealing with the patient's emotional and family problems” or “offering a detailed explanation to patient about his/her potential problems” or “enabling patients or active participation in making any decision or care plan” so that less than 10% of the participants had chosen “strongly agree” or “always” choices in these questions.

**Table 2:** The family physicians’ knowledge and practice towards doctor- patient communication

**Knowledge Questions**	**Strongly agree**	**Agree**	**Neutral**	**Disagree**	**Strongly disagree**
1-When initiating a visit, the physician should greet the patient	310 (77.5)	88 (22.0)	0 (0.0)	0 (0.0)	2 (0.5)
2-In baseline visit, the physician should introduce himself/herself to the patient	279 (69.8)	114 (28.5)	7 (1.8)	0 (0.0)	0 (0.0)
3- At the start of interview, asking open-ended, non-directive questions, encourages patient to tell the story of the problem(s)	271 (67.8)	126 (31.5)	2 (0.5)	1 (0.3)	0 (0.0)
4- Physician should actively listen to and avoid interrupting patients before they could express their concerns	246 (61.5)	147 (36.8)	6 (1.5)	1 (0.3)	0 (0.0)
5- Maintaining consistent eye contact with the patient shows that the doctor is attentive towards his/her patient	143 (35.8)	235 (58.8)	18 (4.5)	4 (1.0)	0 (0.0)
6- Physician should acquiesce to patient request for unnecessary tests when the patient is not convinced by a rational argumentation (reverse question)	19 (4.8)	65 (16.3)	64 (16.0)	177 (44.3)	75 (18.8)
7-Physician is usually unable to resolve patient's emotional, family and social problems and therefore should not deal with such issues (reverse question)	0(0.0)	3(0.8)	109(27.3)	216(54.0)	72(18.0)
8-The physician should encourage patients to express their expectations, so that they could comfortably address their information needs	41(10.3)	241(60.3)	112(28.0)	5(1.3)	1(0.3)
9-Physician should display emotional reactions to the patient's intense feelings such as sadness or stress and express regret over the patient's problems. (reverse question)	1(0.3)	6(1.5)	147(36.8)	216(54.0)	30(7.5)
10-Physician should enable the patient to actively participate in the decision-making process	32 (8.0)	207 (51.7)	140 (35.0)	21(5.3)	0 (0.0)
**Practice questions**	**Always**	**Often**	**Occasionally**	**Seldom**	**Never**
1- When I meet a patient, I'll try to greet him/her first	289 (72.3)	108 (27.0)	3 (0.8)	0 (0.0)	0 (0.0)
2- When I meet patients for the first time, I'll introduce myself	292 (73.0)	79 (19.8)	26 (6.5)	3 (0.8)	0 (0.0)
3- When starting a visit, I prefer to ask open-ended questions rather than closed ones	287 (71.8)	107 (26.8)	4 (1.0)	0 (0.0)	2 (0.5)
4- I’ll interrupt a talkative patient who is wasting my time. (reverse question)	21 (5.3)	37 (9.3)	150 (37.5)	111(27.8)	81 (20.3)
5-I maintain eye contact with the patient throughout the interview	208(52.0)	173(43.3)	16(4.0)	1(0.3)	2(0.5)
6- When patients ask for unnecessary test(s), I'll negotiate to understand and resolve their concerns and try to discourage them by explaining that the requested test(s) yields little or no value	67 (16.8)	268 (67.0)	58 (14.5)	6 (1.5)	1 (0.3)
7- I will not deal with the patient's emotional and family problems, because I am not able to resolve such problems. (reverse question)	70(17.5)	130(32.5)	109(27.3)	76(19.0)	15(3.8)
8-I am too busy and don’t have time to offer a detailed explanation to patient about his/her potential problems (reverse question)	15(3.8)	60(15.0)	51(12.8)	160(40.0)	114(28.5)
9-I react to patient's intense feelings such as sadness and express my emotions. (reverse question)	37(9.3)	131(32.8)	151(37.8)	74(18.5)	7(1.8)
10-I ask my patient to actively participate in making any decision or care plan	32(8.0)	214(53.5)	144(36.0)	10(2.5)	0(0.0)

Table 3 presents the result of association among knowledge & practice scores with demographic and professional variables. Mean knowledge score of female family physicians was higher than that of their male counterparts; and this difference was statistically significant (*P *= 0.02). Mean knowledge score was 42.4 ± 3.4 among family physicians who work in private setting and 41.3 ± 2.6 among those who work in government /public settings (*P *= 0.01). In practice scale, mean practice scores were 38.9 ± 3.5 and 38.0 ± 3.4 among family physicians who worked in public and private settings, respectively, and this difference was statistically significant (*P *= 0.04).

**Table 3:** Mean of knowledge and practice scores towards doctor- patient communication by categorical demographic and professional data

Variables	Knowledge score	Practice score
	Mean ± SD	Mean ± SD
**Gender**		
Men	41.21 ± 3.20	38.73 ± 3.93
Women	41.86 ± 2.29	38.66 ± 2.89
*p-value*	0.020	0.835
**Practice setting**		
Governmental /public	41.31 ± 2.58	38.87 ± 3.47
Private	42.37 ± 3.44	38.01 ± 3.35
*p-value*	0.010	0.044

Results of correlations between knowledge & practice scores and numerical variables are shown in Table 4. According to the Pearson rank correlation coefficient, there was a significant positive correlation between mean knowledge and practice scores (r: 0.309, *P *< 0.001). Also, there were negative correlations between knowledge scores with physicians' age (r: -0.017; *P *< 0.730) and work experience (r:—0.004; *P *< 0.937), both of which were not statistically significant.

**Table 4:** Correlation between age and work experience with participants’ scores for knowledge and practice

Variables	Analysis	Age, y	Work experience	Knowledge score	Practice score
**Age**	Correlation coefficient	1			
	*p- value*	-			
**Work experience**	Correlation coefficient	0.961	1		
	*p- value*	0.000	-		
**Knowledge score**	Correlation coefficient	-0.017	-0.004	1	
	*p- value*	0.730	0.937	-	
**Practice score**	Correlation coefficient	0.137	0.096	0.309	1
	*p- value*	0.006	0.055	0.000	-

We found significant positive linear correlations between mean practice scores and physicians' age (r: 0.137; *P *< 0.006). Correlation between work experience and mean practice scores was positive and borderline significant (r: 0.096; *P *< 0.055).

Results of both univariate and multivariate linear regression analysis of independent variables showed that Iranian family physicians, self-reported practices of doctor- patient communication skills were positively associated with higher ages (and accordingly longer work experience) (β** = **0.06, 95% CI: 0.01 to 0.10, *P *= 0.004) and their related knowledge (β** = **0.41, 95% CI: 0.218 to 0.528, *P *< 0.001) and were negatively associated with working in private setting (β**=**1.24, 95% CI:—2.048 to—0.442, *P *= 0.005). (Table 5).

**Table 5:** Predictors of Iranian family physicians, self-reported practices with regard to doctor- patient communication skills using univariate and multivariate linear regression

**Variables**	**Univariate**			**Multivariate**		
	**β**	**95% CI**^a^**for β**	**P value**	**β**	**95% CI for β**	***P* value**
Age	0.06	0.01 to 0.10	0.006	0.06	0.01 to 0.10	0.004
Sex (Female)	- 0.07	- 0.75 to 0.61	0.836	- 0.09	- 0.74 to 0.55	0.780
Practice setting (Private office)	- 0.80	- 1.70 to -0.02	0.044	- 1.24	- 2.04 to—0.44	0.005
Knowledge score	0.13	0.26 to 0.49	< 0.001	0.41	0.21to 0.52	< 0.001

^a^*CI* Confidence interval

## Discussion

Acquiring professional competence in communication skills may be more critical for family physicians than other healthcare workers, because they spend more time with patients and are more commonly engaged in preventive practices and collaborative decision making. Good doctor-patient relationship allows for better understanding of patient problems and fosters greater patient satisfaction and facilitate patient behavior changes [5, 25].

Our results revealed that most of family physicians had a good level of knowledge regarding doctor- patient communication skills, however, they reported insufficient practice of these skills. It seems that knowledge of family physicians did not fully translate into practice and the problem still exists. Poor communication skills among physicians in different levels of care has been reported in several prior studies [4, 16–18, 21]. Sun et al. [14] in 2016 evaluated 7 primary care physicians in 182 consultations. They reported poor performance of physicians in their interviews. Their participants’ performance was more based on their personality/experience than their knowledge. This indicates that the concept of doctor-patient communication, as a set of learned skills, need to be taught more effectively, beside the positive attitude, desire to learn and self- efficacy.

More detailed analysis of frequency of the participants’ responses to knowledge and practice questions shows that most family physicians were not strongly confident in some important topics. All of these issues including “dealing with the patient's emotional and family problems”, “offering a detailed explanation to patient about his/her potential problems”, “reacting to patient's intense feelings such as sadness or stress and expressing emotions” and “enabling patients or active participation in making any decision or care plan” are important items of Calgary-Cambridge Guides and patient- centered care models [1]. Patients may have different needs including physical, psychological, social and spiritual ones that should be identified and addressed by family physicians through active listening to patients and appropriate gathering of relevant information. This issue is considered as a key element of patient-centered care [27]. More importantly, considering patient context factors (goals, values and expectations) and preferences in care plan is essential part of formulating a solution in all models and guidelines [1, 27, 28]. Al- Zahrani et al. in 2015 [29] reported that less than 10% of general practitioners always involved the patient in decision making or discussed goals of consultation with their patients. Knowledge/ practice gap in each step of communication can be family physician’s challenge and leads to ineffective care. Therefore, increasingly, family physicians should be knowledgeable of the evidence- based guides and models.

Although in univariate analysis, family physicians who worked in private offices had higher mean scores of knowledge and lower practice scores compared with those of the public sector, results of the multivariate regression, controlled for physicians, knowledge, indicated that Iranian family physicians, self-reported practices of doctor- patient communication skills were negatively associated with working in private setting. A study conducted in Saudi Arabia showed that public physicians provoke higher levels of patient trust compared to physicians who work in private sectors, which may be the result of better physicians’ communication competencies in this setting [30]. Similar findings have also been reported from Cambodia and South Australia [31, 32]

In terms of participants’ age and work experience, having higher age and longer work experience were positively associated with higher levels of self-reported practices. Our results agreed with previous reports of Hydarzade et al. and Al-Zahrani et al. [18, 29]. Higher professional experience and presence in the community could lead to better practice.

Moderate to poor practice scores among Iranian family physicians might be explained by cultural norms of patient- physician interactions in Iran, as an Asian country. Intercultural differences in communication style of physicians have been reported in previous studies. For example, in a study conducted by Matusitz et al., comparison of the doctor-patient communication styles of American physicians with those of three Asian countries including Pakistan, Japan, and Thailand showed major dissimilarities between communicative manner of United States' physicians and those of Asian countries, so that Asian doctors were more authoritative and did much of the talking. The Asian countries, physicians shared some similarities in different aspects of the doctor-patient relationship such as religious or philosophical views to the health care, paternalistic approach to patients and collectivistic and scripted styles of communication [33]. In a qualitative study, perceptions of physicians from a Western country (Sweden) were assessed regarding physician–patient communication after watching a video from the Iranian context. According to findings of this study, Iranian patients were not convenient during history taking due to exaggerated respect to doctor that might be related to cultural beliefs about Physicians' social class. Most Iranian doctors did not look at or listen to their patients during medical visit and it seemed that they were just asking a list of prepared questions. Also, they did not consider shared decision making with patients. Participants’ perceptions and comparison with their own work experiences indicated major inter-cultural and cross-cultural differences [34]. Obviously, a culture change takes time and involves a reframing of norms and expectations within the health care-associated organizations and in the society. Finally, we should emphasize on periodic refreshing courses on standard doctor-patient communication skills for all health professionals, particularly family physicians.

## Strengths and limitations

To the best of our knowledge, this is the first study of doctor- patient communication knowledge and practice among urban family physicians in Iran. As large number of sample size, the results provide valid information for managers. The project was presented as an educational needs assessment, participants did not feel that they were being examined, so response rates were excellent. This study was not free from limitations. First, our participants were recruited from urban family physicians affiliated to Shiraz University of Medical Sciences and therefore might not be representative of all Iranian urban family physicians. Second limitation was small number of publications on this target population, which made comparisons difficult and third, we did not measure participants’ attitude toward communication skills which may affect their practice.

## Conclusion

There is a potential gap between knowledge and self-reported practices toward communication skills among a sample of Iranian family physicians and it seems that a high level of knowledge does not guarantee good practice. They have fundamental weakness in the most important evidence-based items of doctor- patient communication. Considering significant role of family physicians in prevention and control of non-communicable diseases (NCDs) as an emerging challenge of our country, the topic of communication skills should be inserted as a top educational priority of family physicians. And it given sufficient weight in objective structured clinical examinations with immediate feedback. In order to knowledge will be fully translate in practice, we need to empower our family physicians with positive attitude and self- efficacy in doctor- patient communication. Further research will be required to evaluate the effectiveness of the new interventions.

## Data Availability

The datasets used and/or analyzed during the current study are available from the corresponding author on reasonable request.

## Abbreviations

NCDs: Non-communicable diseases
CI: Confidence interval

## References

[1] Berman AC, Chutka DS (2016). Assessing effective physician-patient communication skills: “Are you listening to me, doc?”. Korean J Med Educ.

[2] Ranjan P, Kumari A, Chakrawarty A (2015). How can doctors improve their communication skills?. J Clin Diagn Res.

[3] Levinson W, Lesser CS, Epstein RM (2010). Developing physician communication skills for patient-centered care. Health Aff (Millwood).

[4] Iversen ED, Wolderslund MO, Kofoed PE, Gulbrandsen P, Poulsen H, Cold S (2020). Codebook for rating clinical communication skills based on the Calgary-Cambridge Guide. BMC Med Educ.

[5] Simmenroth-Nayda A, Heinemann S, Nolte C, Fischer T, Himmel W (2014). Psychometric properties of the Calgary Cambridge guides to assess communication skills of undergraduate medical students. Int J Med Educ.

[6] Ha JF, Longnecker N (2010). Doctor-patient communication: a review. Ochsner J.

[7] Ghaffarifar S, Ghofranipour F, Ahmadi F, Khoshbaten M (2015). Barriers to Effective Doctor-Patient Relationship Based on PRECEDE PROCEED Model. Glob J Health Sci.

[8] Constand MK, MacDermid JC, Dal Bello-Haas V, Law M (2014). Scoping review of patient-centered care approaches in healthcare. BMC Health Serv Res.

[9] Chipidza FE, Wallwork RS, Stern TA. Impact of the Doctor-Patient Relationship. Prim Care Companion CNS Disord. 2015;17(5):10.4088/PCC.15f01840. 10.4088/PCC.15f01840. 10.4088/PCC.15f01840 PMC4732308 26835164

[10] Harbishettar V, Krishna KR, Srinivasa P, Gowda M (2019). The enigma of doctor-patient relationship. Indian J Psychiatry.

[11] Ishikawa H, Hashimoto H, Kiuchi T (2013). The evolving concept of “patient-centeredness” in patient-physician communication research. Soc Sci Med.

[12] Peykari N, Hashemi H, Dinarvand R, Haji-Aghajani M, Malekzadeh R, Sadrolsadat A (2017). National action plan for non-communicable diseases prevention and control in Iran; a response to emerging epidemic. J Diabetes Metab.

[13] Aminorroaya A, Fattahi N, Azadnajafabad S, Mohammadi E, Jamshidi K, Khalilabad MR, et al. Burden of non-communicable diseases in Iran: past, present, and future. J Diabetes Metab Disord. 2020. 10.1007/s40200-020-00669-z. 10.1007/s40200-020-00669-z PMC11599516 39610553

[14] Sun N, Rau P-LP (2017). Barriers to improve physician–patient communication in a primary care setting: perspectives of Chinese physicians. Health Psychol Behav Med.

[15] Kapil M, Verma A, Sareen R. Communication skills in medicine-an art of learning. EC Clin Med Case Rep. 2019;(2.6):281–3.

[16] Rezaeian M, Zare-Bidaki M, Bakhtar M, Kargar S (2015). A survey on communication skills of Rafsanjan University of Medical Sciences Faculty Members in 2013. JRUMS.

[17] Bagheri R, Mohammadikia SA, Kazemian M, Kazemi B, Rezapour M (2014). Satisfaction rate from the communication skills of family physicians in rural health centers at Mazandaran University of medical sciences. JRUMS.

[18] Heidarzadeh A, Dadkhah Tirani H, Asadi A, Nemati MJRiME. The Interns, residents and faculty members' knowledge and attitudes toward communication skills. RME. 2007;9:26–31.

[19] Shukla AK, Yadav VS, Kastury N (2010). Doctor-patient communication: an important but often ignored aspect in clinical medicine. JIACM.

[20] Liu X, Rohrer W, Luo A, Fang Z, He T, Xie W (2015). Doctor–patient communication skills training in mainland China: a systematic review of the literature. Patient Educ Couns.

[21] Ghaffarifar S, Khoshbaten M, Ghofranipour F, Kompani JJSE-MJ. Residents’ Communication skills evaluation by OSCE: What changes should be done in educational system. Shiraz E Med J. 2013;14(4).

[22] Dhand NK, & Khatkar, M. S. . Statulator: An online statistical calculator. Sample Size Calculator for Estimating a Single Proportion. 2014.

[23] Main CJ, Buchbinder R, Porcheret M, Foster NJBP, Rheumatology RC (2010). Addressing patient beliefs and expectations in the consultation. Best Pract Res Clin Rheumatol.

[24] Burt J, Abel G, Elmore N, Campbell J, Roland M, Benson J (2014). Assessing communication quality of consultations in primary care: initial reliability of the global consultation rating scale, based on the Calgary-Cambridge guide to the medical interview. BMJ Open.

[25] Burt J, Abel G, Elmore N, Newbould J, Davey A, Llanwarne N (2018). Rating communication in GP consultations: the association between ratings made by patients and trained clinical raters. Med Care Res Rev.

[26] Hwaid AH (2013). Knowledge and awareness of papillomavirus and cervical cancer among college students and health care workers women in Diyala Iraq. Am J Public Health Res..

[27] Rakel R. Clinical Problem Solving. Textbook of Family Medicine. 9th ed. Philadelphia: Elsevier; 2016. p. 1215.

[28] Murtagh J JR, Coleman J, Murtagh C. John Murtagh’s General Practice. 7th ed. Australia: Michael Weitz; 2018. p. 19-27.

[29] Al-Zahrani BS, Al-Misfer MF, Al-Hazmi AM (2015). Knowledge, attitude, practice and barriers of effective communication skills during medical consultation among general practitioners national guard primary health care center, Riyadh Saudi Arabia. MEJFM.

[30] Aljaffary A, AlThumairi A, Almarhoon L, Alsaawi G (2021). Measuring patient trust in public versus private physicians in the Kingdom of Saudi Arabia (KSA). J Multidiscip Healthc.

[31] Ozawa S, Walker DG (2011). Comparison of trust in public vs private health care providers in rural Cambodia. Health Policy Plan.

[32] Ward PR, Rokkas P, Cenko C, Pulvirenti M, Dean N, Carney S (2015). A qualitative study of patient (dis)trust in public and private hospitals: the importance of choice and pragmatic acceptance for trust considerations in South Australia. BMC Health Serv Res.

[33] Matusitz J, Spear J (2015). Doctor-patient communication styles: A comparison between the United States and three Asian countries. J Hum Behav Soc Environ.

[34] Shirazi M, Ponzer S, Zarghi N, Keshmiri F, Motlagh MK, Zavareh DK (2020). Inter-cultural and cross-cultural communication through physicians’ lens: perceptions and experiences. Int J Med Educ.

